# "Shock and kill" effects of class I-selective histone deacetylase inhibitors in combination with the glutathione synthesis inhibitor buthionine sulfoximine in cell line models for HIV-1 quiescence

**DOI:** 10.1186/1742-4690-6-52

**Published:** 2009-06-02

**Authors:** Andrea Savarino, Antonello Mai, Sandro Norelli, Sary El Daker, Sergio Valente, Dante Rotili, Lucia Altucci, Anna Teresa Palamara, Enrico Garaci

**Affiliations:** 1 Dept of Infectious, Parasitic and Immune-mediated Diseases, Istituto Superiore di Sanità, Viale Regina Elena, 299, 00161, Rome, Italy; 2 Pasteur Institute, Cenci-Bolognetti Foundation, Dept of Drug Chemistry and Technologies, Sapienza University of Rome, P.le A. Moro, 5, 00185, Rome, Italy; 3 Dept of General Pathology, 2nd University of Naples, Vico L. De Crecchio 7, 80138 Naples, Italy; 4 Pasteur Institute, Cenci-Bolognetti Foundation, Dept of Public Health Sciences, Sapienza University of Rome, P.le A. Moro, 5, 00185, Rome, Italy; 5 Dept of Experimental Medicine, University of Rome Tor Vergata, Rome, Italy; 6 IRCCS San Raffaele Pisana, via della Pisana 235, 00163 Rome, Italy

## Abstract

Latently infected, resting memory CD4^+ ^T cells and macrophages represent a major obstacle to the eradication of HIV-1. For this purpose, "shock and kill" strategies have been proposed (activation of HIV-1 followed by stimuli leading to cell death). Histone deacetylase inhibitors (HDACIs) induce HIV-1 activation from quiescence, yet class/isoform-selective HDACIs are needed to specifically target HIV-1 latency. We tested 32 small molecule HDACIs for their ability to induce HIV-1 activation in the ACH-2 and U1 cell line models. In general, potent activators of HIV-1 replication were found among non-class selective and class I-selective HDACIs. However, class I selectivity did not reduce the toxicity of most of the molecules for uninfected cells, which is a major concern for possible HDACI-based therapies. To overcome this problem, complementary strategies using lower HDACI concentrations have been explored. We added to class I HDACIs the glutathione-synthesis inhibitor buthionine sulfoximine (BSO), in an attempt to create an intracellular environment that would facilitate HIV-1 activation. The basis for this strategy was that HIV-1 replication decreases the intracellular levels of reduced glutathione, creating a pro-oxidant environment which in turn stimulates HIV-1 transcription. We found that BSO increased the ability of class I HDACIs to activate HIV-1. This interaction allowed the use of both types of drugs at concentrations that were non-toxic for uninfected cells, whereas the infected cell cultures succumbed more readily to the drug combination. These effects were associated with BSO-induced recruitment of HDACI-insensitive cells into the responding cell population, as shown in Jurkat cell models for HIV-1 quiescence. The results of the present study may contribute to the future design of class I HDACIs for treating HIV-1. Moreover, the combined effects of class I-selective HDACIs and the glutathione synthesis inhibitor BSO suggest the existence of an Achilles' heel that could be manipulated in order to facilitate the "kill" phase of experimental HIV-1 eradication strategies.

## Findings

Given the inability of antiretroviral therapy (ART) to eradicate HIV-1 from the body (even after decade-long periods of therapy), and the absence of effective vaccines on the horizon, novel approaches to HIV-1 eradication are needed. To this end, the so-called "shock and kill" strategies have been proposed [1]. These strategies consist of inducing, through drugs, HIV-1 activation from quiescence (*i.e. *the "shock" phase), in the presence of ART (to block viral spread), followed by the elimination of infected cells (*i.e. *the "kill" phase), through either natural means (e.g. immune response, viral cytopathogenicity) or artificial means (*e.g. *drugs, monoclonal antibodies, etc.) [1]. For the "shock" phase, histone deacetylase inhibitors (HDACIs) have been proposed [2]. Histone deacetylases (HDACs) contribute to nucleosomal integrity by maintaining histones in a form that has high affinity for DNA [3]. Physiologically, this activity is counteracted by histone acetyl transferases (HATs) which are recruited to gene promoters by specific transcription factor-activating stimuli [3].

Several of the currently available HDACIs activate HIV-1 from quiescence *in vitro *[4,5]. However, this activity is associated with a certain degree of toxicity [6], given that these inhibitors are not class-specific and compromise a large number of cellular pathways [7,8]. Class I HDACs comprise HDAC1-3 and 8; they are predominantly nuclear enzymes and are ubiquitously expressed [9]. Class II HDACs include HDAC4-7, 9 and 10 and shuttle between the nucleus and the cytoplasm [10,11]. HDACs are recruited to the HIV-1 promoter by several transcription factors, including NF-κB (p50/p50 homodimers), AP-4, Sp1, YY1 and c-Myc [12-14]. Identification of class/isoform-selective HDACIs with increased potency and lower toxicity [3] and drugs able to potentiate their effects is believed to be important for HIV-1 eradication.

To identify novel HDACIs capable of activating HIV-1, we first tested the HIV-1 activating ability of our institutional library of HDACIs [see Additional file 1] in cell lines in which HIV-1 is inducible (*i.e. *T-lymphoid ACH-2 cells and monocytic U1 cells). The potency of these molecules to activate HIV-1 was assessed in terms of p24 production, as measured by ELISA (Perkin-Elmers, Boston, MA), following incubation with a drug concentration of 1 μM (generally used as a threshold for selection of lead compounds). As a positive control, we used TNF-α (5 ng/ml), a cytokine that activates HIV-1 transcription through NF-κB (p65/p50) induction [1]. As a reference standard for the comparison of results, we used suberoylamide hydroxamic acid (SAHA; also referred to as "vorinostat"), a non-specific inhibitor of both classes of HDACs when used in the upper-nanomolar/micromolar range of concentrations [15].

**Figure 1:**
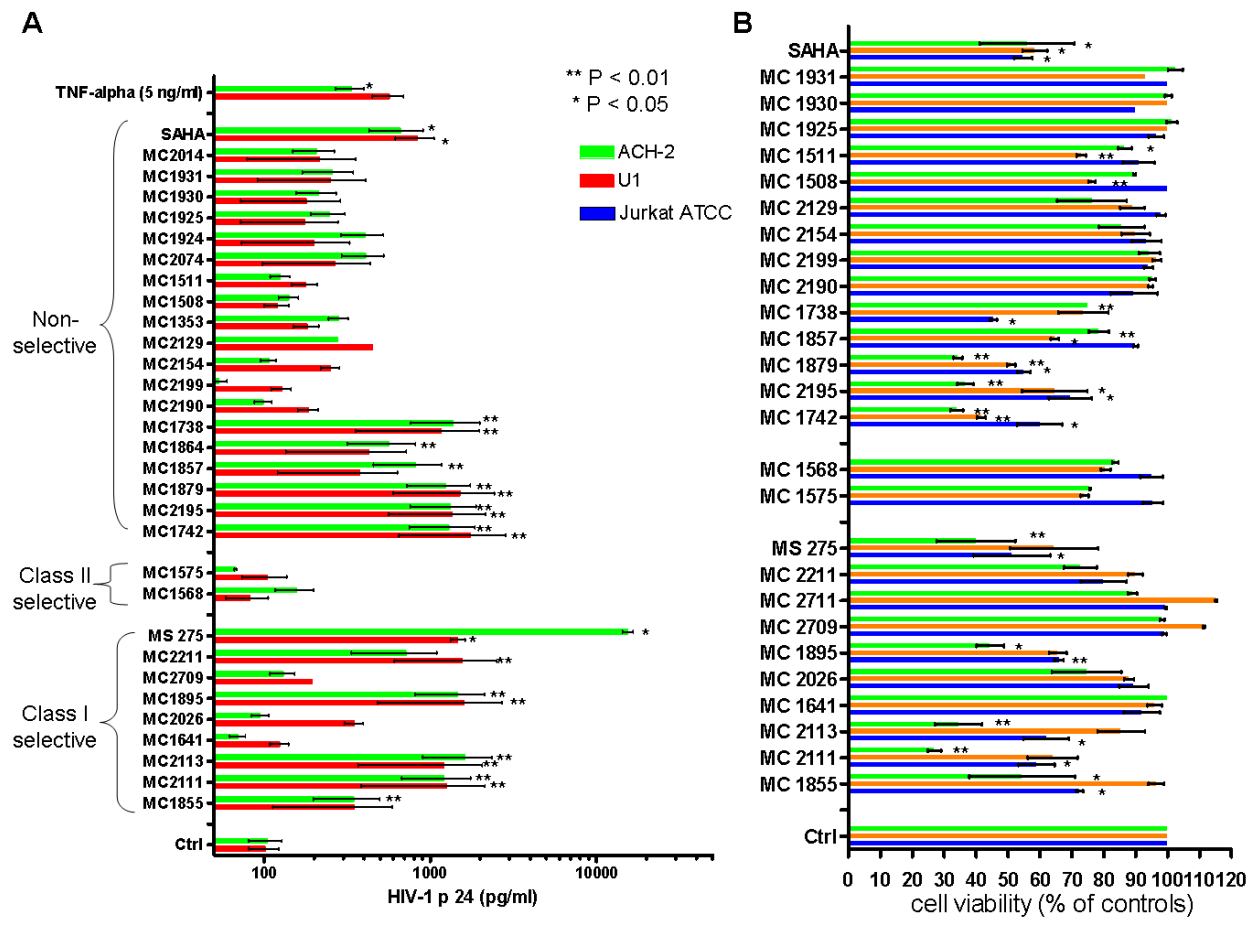
**Potencies of different HDACIs in terms of activation of HIV-1 replication in U1 and ACH-2 cells, and toxicity in uninfected Jurkat T-cells**. Panel *A*: Cells were incubated with the test compounds (1 μM), and p24 production was measured by ELISA in cell culture supernatants at 72 hours post-infection (means ± SEM; 3 experiments). Asterisks show the significant differences in comparison to untreated control cultures according to repeated-measures ANOVA using Dunnet's multiple comparison post-test (a *Log *transformation of p24 values was applied to restore normality). Panel *B*: Uninfected Jurkat T cells were incubated for 72 h under similar conditions, and toxicity was measured by the methyl tetrazolium (MTT) method. Results are presented as a percentage of the O.D. (λ = 550) of untreated controls subtracted of background (means ± SEM; 3 experiments). Asterisks show the significant differences in comparison to untreated control cultures according to repeated-measures ANOVA using Dunnet's multiple comparison post-test.

**Figure 2:**
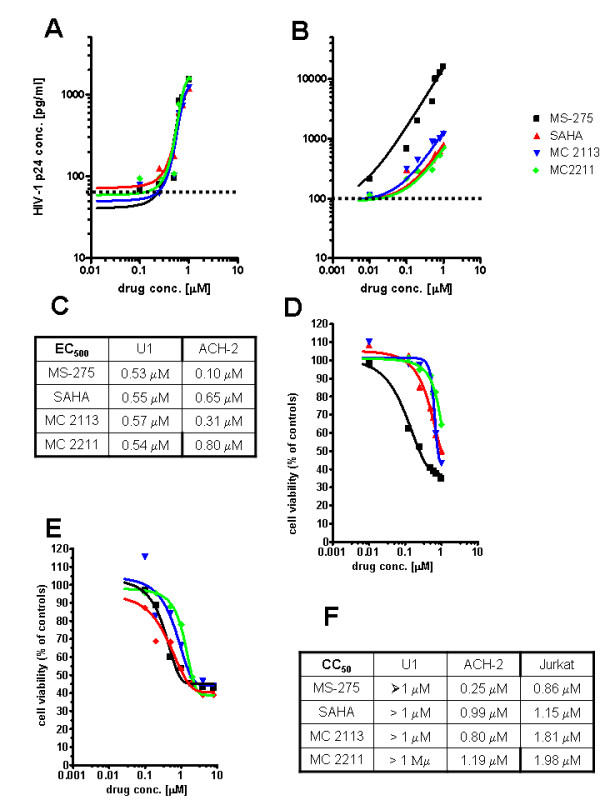
**Dose-dependent activation of HIV-1 replication by class I-selective HDACIs and corresponding toxicity in U1 and ACH-2 cells**. Panels *A, B*: Concentration-dependent stimulation of HIV-1 p24 production in the latently infected cell lines U1 (*A*) and ACH-2 (*B*) at 72 hours of incubation with MS-275, MC2211, MC2113 (class I-selective HDACIs) and SAHA (a non-class-selective HDACI used as a positive control). Mean values are from three independent experiments (error bars are not shown for better clarity). Dotted lines represent the average p24 levels found in untreated controls in the same experiments. Panel *C. *Effective concentrations for increasing viral replication to 500% of the basal levels of untreated controls (EC_500_). Panel *D*: Cell viability of ACH-2 cells, as measured by the methyl tetrazolium (MTT) method. Results are presented as a percentage of the O.D. (λ = 550) of untreated controls subtracted for background (means ± SEM; 3 experiments). Panel *E*: Cell viability of uninfected Jurkat T cells incubated for 72 hours with the same drugs is shown as comparison. Panel *F. *50% cytotoxic concentrations (CC_50_). For the symbols in panels *D, E*, the reader should refer to those of panels *A, B*.

The results revealed a number of compounds capable of activating HIV-1; and, for the most potent compounds, there was good agreement between the results in the ACH-2 and U1 cells (Figure 1). Only non-class selective and class I-selective HDACIs were significantly active (Figure 1), and potent class I-selective HDACIs enhanced HIV-1 replication in the nanomolar range in a dose-dependent manner (Figure 2). In general, class I selectivity was insufficient for eliminating toxicity, although some of the compounds (*e.g. *MC2211) induced adequate HIV-1 activation and low-level toxicity (Figure 1, 2). Of note, the class I-selective HDACIs that activated HIV-1 included MS-275, an HDAC1-3-selective inhibitor currently being tested in phase II clinical trials as an anticancer drug [15].

A previous study showed a trend towards higher toxicity of the HDACI trichostatin in ACH-2 cells than in their uninfected counterparts and linked this phenomenon to the cytotoxicity of activated HIV-1 replication in lymphoid cells [16]. In our experiments, three different class I HDACIs (*i.e*. MS-275, MC2113 and MC2211) displayed lower CC_50 _in ACH-2 cells (Figure 2D) than in uninfected CD4^+ ^T cells (data from Jurkat cells are shown as an example in Figure 2E), yet the extent of the difference did not support the possibility of a "therapeutic window". The same compounds displayed non-significant toxicity in U1 cells at concentrations up to 1 μM (Figure 2F).

In these experiments, an incubation period of 72 hours was preferred to shorter periods, because of the intrinsically slow mode of action of epigenetic modulators, which only indirectly induce HIV-1 activation. This was confirmed by our experiments using Jurkat cell clones with an integrated green fluorescence protein (GFP)-encoding gene under control of the HIV-1 LTR [17]. In these Jurkat cell clones, GFP induction by HDACIs was evident only in a fraction of cells at 24 hours of incubation and increased over time [see Additional file 2].

**Figure 3:**
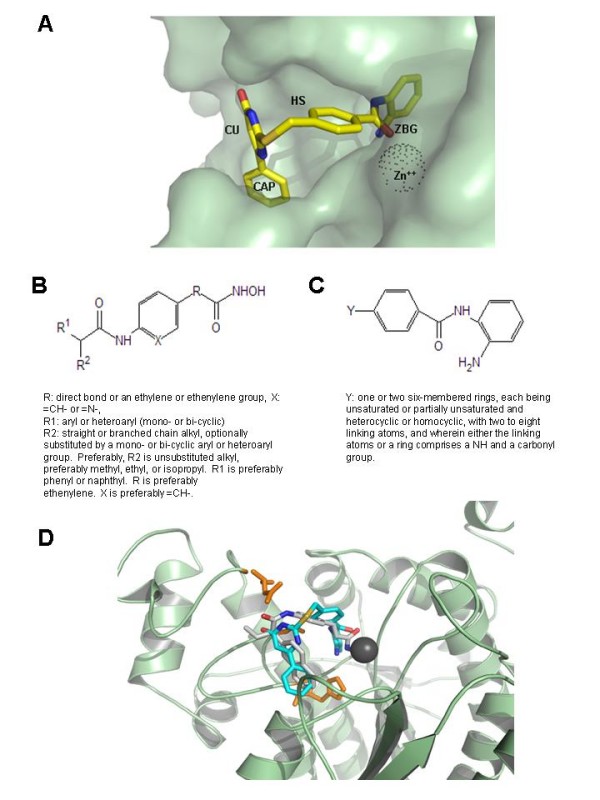
**Structural characteristics of HIV-1 activating HDACIs**. Panel A: Docking of the HDACI MC2211 at the catalytic cavity of HDAC2, a class I enzyme. The different portions of the inhibitor [*i.e. *the CAP portion (CAP), the connection unit (CU), the hydrophobic spacer (HS), and the zinc-binding group (ZBG)] are mapped to the molecule represented in the picture. The enzyme is shown as semi-transparent Connolly surface. The Zn^++ ^ion embedded in the catalytic cavity is shown as a dotted sphere. The inhibitor is shown according to CPK colouring. Panels B, C: General formulas for HDACIs capable of inducing HIV-1 activation from quiescence. Panel D: Structural superimposition of the best docking poses for the HDACIs MC2113 and MC2211 within the catalytic cavity of HDAC2. Inhibitors are shown in CPK (MC2113: carbon backbone in white; MC2211: carbon backbone in cyan). The enzyme backbone is shown as cartoons. The Zn^++ ^ion is shown as a gray sphere. Amino acids D100, H141 and G150 (important for hydrogen bonding with the inhibitors) are shown as orange sticks.

To focus on the structural requirements for the most potent class I-selective HDACIs, we then performed a structure/activity relationship (SAR) study. SAR studies relate the effect or the potency of bioactive chemical compounds with their chemical structure and help to understand the structural requirements for obtaining a desired effect. HDACIs are structured according to a general pharmacophore model (*i.e*. "a molecular framework that carries the essential features responsible for a drug's biological activity" [18]) (Figure 3A). This pharmacophore model comprises a cap group (CAP), a polar connection unit (CU), and a hydrophobic spacer (HS), which carries at its end a Zn^2+ ^binding group (ZBG), able to complex the Zn^2+ ^at the bottom of the cavity [19]. The ZBG consists of a hydroxamate, a sulfhydryl, or a benzamide moiety (Figure 3A shows a benzamide inhibitor complexed with HDAC2). A general scaffold describing the characteristics of the most potent HDACIs from our library is presented in Figure 3B, C. The differences in the general structural requirements for the two main chemical types of HDACIs in our library (hydroxamates and benzamides) can probably be attributed to the hydrophobicity/hydrophilicity balance (the more hydrophobic benzamides require less hydrophobic CAP groups than hydroxamates do). The molecular docking simulations, conducted as previously described [20,21], highlighted particular requirements for the CU (Figure 3D). These requirements consisted of a uracil group or an amide group in a *cis*-conformation, which presented the nitrogen-bond hydrogen and the carbonylic oxygen on the same side of the molecule (usually amide groups are in a *trans*-conformation, with the nitrogen-bond hydrogen and the carbonylic oxygen oriented in opposite directions) (Figure 3D). SAHA, consistent with its non-specific inhibitory activity on HDACs [15], did not match the characteristics of our pharmacophore model [see Additional file 3].

Given that class I selectivity, in general, did not markedly decrease the toxicity of HDACIs, we have begun studies on complementary strategies that might increase the efficacy of class I HDACIs at non-toxic concentrations. It is well known that HIV-1 induces a pro-oxidant status which in turn enhances the levels of HIV-1 transcription [22-25]. There are probably many mechanisms behind HIV-1-induced oxidative stress, and the signals that it sparks are still far from being fully understood [26]. In general, oxidative stress tilts the balance of HAT/HDAC activity towards increased HAT activity and DNA unwinding, thus facilitating the binding of several transcription factors [27]. The HIV-1-induced pro-oxidant status is in part mediated by decreased intracellular levels of reduced glutathione [26,28]. The depletion of reduced glutathione has been linked to activation of viral replication [29], whereas the administration of this cofactor results in antiretroviral effects [26]. We hypothesized that glutathione depletion might create an intracellular environment that facilitates HIV-1 activation by HDACIs. To test this hypothesis, we evaluated the HIV-1 activating effects of buthionine sulfoximine (BSO), which depletes glutathione by inhibiting γ-glutamyl cysteine synthetase (a limiting step in glutathione synthesis) [27,30].

**Figure 4:**
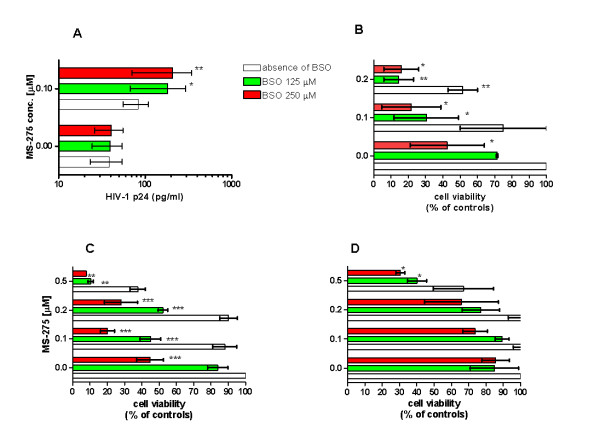
**Effects on HIV-1 replication and cell viability of class I-selective HDACIs, MS-275 and buthionine sulfoximine (BSO), alone or in combination**. Panel *A*: HIV-1 p24 concentrations in ACH-2 cell culture supernatants at 24 hours of incubation with the drugs. Panels *B-D*: Cell viability values at 72 hours of incubation, as determined by the methyl tetrazolium (MTT) method: ACH-2 cells (B), Jurkat 6.3 cells (C), uninfected Jurkat cells (D). Results are presented as percentages of the absorbance (λ = 550) in untreated controls subtracted for background (means ± SEM; 3 experiments). Asterisks show the significant differences found between BSO treatments and matched treatments in the absence of BSO (* *P *< 0.05; ** *P *< 0.01; *** *P *< 0.001). Statistical significance was calculated using repeated-measures, two-way ANOVA and Bonferroni's post-test, following an appropriate transformation to restore normality, where necessary. The higher drug concentrations adopted in Panels C, D serve as comparisons with the experiment in Figure 5.

BSO, at concentrations of up to 500 μM, did not significantly raise the p24 concentrations; yet it increased the HIV-1 promoting effects of class I HDACIs, such as MS-275 (Figure 4A) and MC2113 (data not shown) in ACH-2 cells (Figure 4A) and U1 cells (data not shown). According to the literature, the concentrations of MS-275 and BSO adopted here are clinically achievable [31,32]. The results shown in Figure 4A are based on a 24 hour incubation time, given the marked cytotoxicity shown by the drug combination in the ACH-2 cells at 72 hours of incubation (Figure 4B). Since HIV-1 replicating cell cultures display decreased levels of reduced glutathione [29], their poor tolerance to an inhibitor of glutathione synthesis is not surprising. This concept is supported by experiments in uninfected Jurkat cells and Jurkat cell clones (6.3 and 8.4), which contain a quiescent HIV-1 genome (with the *GFP *gene) under control of the LTR [17]. We found that the 6.3 cell clone succumbed more readily to the MS-275/BSO combination than its uninfected counterpart (Figure 4C, D). Similar results were obtained with the 8.4 clone (data not shown).

**Figure 5:**
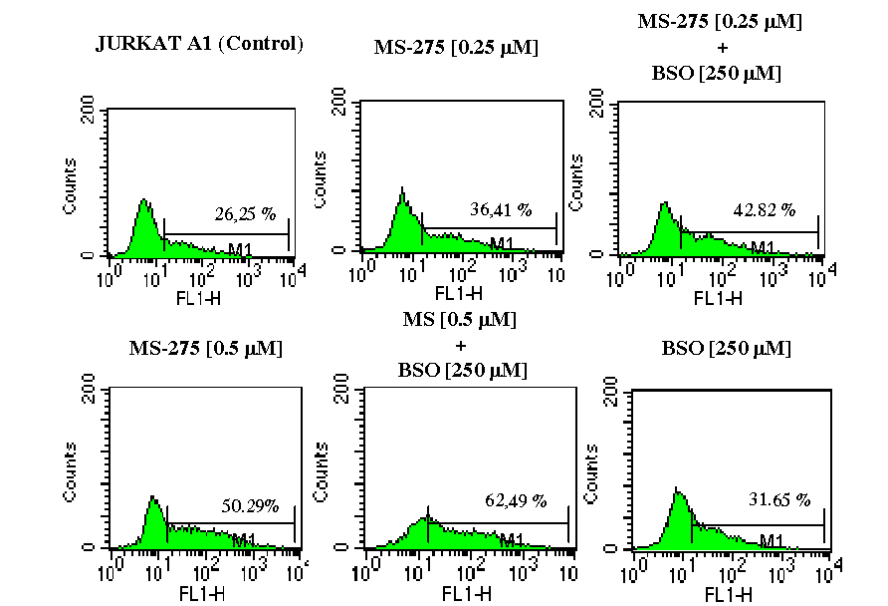
**Stimulation of HIV-1 LTR-controlled expression of green fluorescent protein (GFP) by MS-275 and buthionine sulfoximine (BSO), alone or in combination in a Jurkat cell clone (A1)**. The A1 cell clone, derived from T-lymphoid Jurkat cells, is a model for latent HIV-1 infection. This clone has an integrated GFP/Tat construct under the control of the HIV-1 LTR and displays a basal proportion of cells expressing GFP, which increase following stimuli activating the HIV-1 promoter. A1 cells were incubated for 72 hours with the different treatments, and GFP expression was monitored by standard flow-cytometric techniques and assessed as the percentage of fluorescent cells (indicated for each histogram) beyond the threshold value established using control non-transfected Jurkat cells. One experiment out of three with similar results is shown. The histograms derived from double-drug treatments were found to be significantly different (*P *< 0.01) from those derived from treatments with a single drug at matched concentrations (Kolmogorov-Smirnoff statistics). Differences between the drug concentrations adopted in this experiment and that in Figure 4A are derived from adjustments due to the different nature of the cell lines adopted.

The Jurkat model for HIV-1 quiescence showed that BSO recruited HDACI-insensitive cells into the responding cell population (Figure 5). These results are derived from the A1 Jurkat cell clone, which has an integrated GFP/Tat construct under control of the HIV-1 LTR, which is quiescent in the majority of cells [17]. This clone was chosen because this type of analysis could not be conducted in the 6.3 or 8.4 clones, since, at 24 hours of incubation with the drugs, these clones displayed only a small proportion of cells expressing GFP, and a correct estimate of the expression of this protein at subsequent time points was biased by the autofluorescence of dying cells. The A1 clone, which does not have a complete HIV-1 genome, was less sensitive to the toxic effects of the MS-275/BSO combination than the 6.3 and 8.4 clones (data not shown).

To sum up, the combination of a class I-selective HDACI and BSO activates HIV-1 at concentrations that show low toxicity in uninfected cells, and it induces cell death in infected cell cultures. These results are consistent with a model in which BSO would favor the HIV-1 activating effects of HDACIs by lowering the intracellular levels of reduced glutathione [30] and would induce the death of infected cells by preventing replenishment of the reduced glutathione pools that are further "consumed" by the virus activated from quiescence [28,29]. If these results are confirmed, the decreased pool of reduced glutathione may become an Achilles' heel of the infected cells, and its manipulation may open new avenues to their elimination.

This strategy will of course require optimization, and several issues still have to be addressed. First, not all of the cells with a quiescent provirus respond to the treatment. A variegated phenotype after activation, with only a fraction of the cell population becoming activated in response to a global signal, was also shown by Jordan *et al. *[17], who attributed this phenomenon to the different local chromatin environments. A thorough investigation of the molecular signals sparked by the BSO/class I-selective HDACI combination (currently in progress in our laboratories) is expected to provide insight into these phenomena. Moreover, the "therapeutic window" (*i.e. *the differential toxicity in uninfected *vs*. infected cells) still needs to be widened. In this regard, the general structural requirements for the HIV-1 activating HDACIs presented in our study, as well as the recent identification of HDAC2 as a potential target for HIV-1 reactivation strategies [33], may represent a good starting point for developing next-generation class I HDACIs with increased selectivity and decreased toxicity. Finally, we are currently searching for novel γ-glutamyl-cysteine synthetase inhibitors acting in the nanomolar range and displaying lower toxicity than BSO in uninfected cells.

The concept to activate provirus transcription to target latency is not new, and several clinical trials have been conducted in the past years along this line, ranging from the administration of IL-2 to the utilization of valproic acid [34-36]. The results of these trials have been largely disappointing so far. Valproic acid, a relatively weak HDACI, was tested in a small clinical trial in combination with antiretroviral therapy intensified with the fusion inhibitor enfuvirtide [35,36], but some more recent studies have failed to show a decay of resting CD4^+ ^T cell infection in individuals under valproic acid treatment for clinical reasons while also receiving standard ART [37]. Our study provides a potentially more powerful approach for the "shock" phase of experimental HIV-1 eradicating strategies and a potential tool for the "kill" phase. Notwithstanding the aforementioned need for amelioration, it is interesting to point out that both MS-275 and BSO have passed class I clinical trials for safety in humans and are therefore ready for testing in animal models. Such testing would be important at a time when no proof-of-concept exists for the "shock and kill" theory. In this regard, even a partial response (*e.g. *a reduction in latently infected cells) would be a valuable indicator of the validity of this approach. The possible efficacy of the "shock and kill" approach is still a matter of debate. For example, a recent study of Jeeninga *et al. *suggests that there are different cellular reservoirs for HIV-1 latency and that each reservoir may require a specific activation strategy [38]. Viral factors, along with cellular factors, may contribute to HIV-1 quiescence, and these factors may not be controlled by strategies using HDACIs.

## Competing interests

AS, AM, ATP, and EG have requested patent rights on several compounds described in the present study and on the MS-275/BSO combination.

## Authors' contributions

AS conceived and coordinated the study, supervised the generation of biological data, conducted the molecular docking, analyzed the data and drafted the manuscript. AM conceived the majority of the structures described in the present study, supervised their synthesis and participated in manuscript drafting. SN and SED conducted the biological testing and contributed to molecular modeling and data analysis. SV, DR, and LA conducted synthesis and development of the HDACi. LA conducted the HDAC inhibitory assays. ATP and EG contributed the idea of using BSO for HIV-1 escape from latency and participated in the experimental planning.

## Supplementary Material

Additional file 1 **Structures and HDAC inhibiting activity of the cited HDACIs.** Where data on human HDACs are unavailable, data on maize HD1-B (homologous with human class I HDACs) and HD1-A (homologous with human class II HDACs), or relevant references, are provided. Click here for file

Additional file 2 **To study the HDACI response in a cell population, we used quiescently infected T-lymphoid Jurkat cell clones.** Two types of cell clones were used: 1) A1, and A2, which have an integrated GFP/Tat construct under control of the HIV-1 LTR; 2) 6.3, and 8.4, which contain the entire HIV-1 genome under control of the LTR and have the GFP gene replacing *nef*. The 6.3 cells display insignificant basal levels of GFP expression. Cells were incubated with the different treatments, and GFP expression was monitored in gated live cells at 12, 24 and 72 hours by standard flow cytometric techniques. Results are presented as fluorescence histograms. Each histogram reports the percentage of fluorescent cells beyond a threshold value established using non-infected Jurkat cells. Click here for file

Additional file 3 **Structural superimposition of MC2211 (carbon backbone in cyan) and SAHA (vorinostat; carbon backbone in yellow) docking at the HDAC2 catalytic site.** SAHA, a non-selective HDACI, displays an amide group in a conformation that does not match that of the class I-selective HDACIs (Figure 3). The other molecular players are displayed in the same fashion as in Figure 3. Click here for file
