# Supplementary material for: "Shock and kill" effects of class I-selective histone deacetylase inhibitors in combination with the glutathione synthesis inhibitor buthionine sulfoximine in cell line models for HIV-1 quiescence
Source: Retrovirology. 2009 Jun 2;6:52. doi: 10.1186/1742-4690-6-52 (PMC2697151; doi:10.1186/1742-4690-6-52)
Supplement: Additional file 1 — Structures and HDAC inhibiting activity of the cited HDACIs. Where data on human HDACs are unavailable, data on maize HD1-B (homologous with human class I HDACs) and HD1-A (homologous with human class II HDACs), or relevant references, are provided. [file 1742-4690-6-52-S1.doc]

**Additional file 1.**

Chemical structures and HDAC inhibitory activity (HDAC1/HDAC4 immunoprecipitate assays, % of inhibition at 5 μM, or maize HD1-B and HD1-A, IC_50_ values) or relative references.

|  |  |  |
| --- | --- | --- |
| MC1353  Reff. 1, 2 | MC1511  Ref. 2 | MC1508  Ref. 2 |
|  |  |  |
| MC1568  Reff. 3, 4 | MC1575  Reff. 3, 4 | MC1855  % inhibition HDAC1: 78.7  % inhibition HDAC4: 0 |
|  |  |  |
| MC2111  % inhibition HDAC1: 73.6  % inhibition HDAC4: 0 | MC2113  % inhibition HDAC1: 64.9  % inhibition HDAC4: 22.4 | MC1742  IC_50_ HD1-B: 4.5 nM  IC_50_ HD1-A: 0.8 nM |
|  |  |  |
| MC2195  % inhibition HDAC1: 46.7  % inhibition HDAC4: 93.1 | MC2190  % inhibition HDAC1: 32.0  % inhibition HDAC4: 64.4 | MC1641  Ref. 5 |
|  |  |  |
| MC1879  % inhibition HDAC1: 97.8  % inhibition HDAC4: 89.7 | MC1818  IC_50_ HD1-B: 8 nM  IC_50_ HD1-A: 7 Nm | MC2211  Ref. 6 |
|  |  |  |
| MC2026  IC_50_ HD1-B: 129 nM  IC_50_ HD1-A: 32 nM | MC2199  % inhibition HDAC1: 48.1  % inhibition HDAC4: 68.1 | MC2154  % inhibition HDAC1: 42.0  % inhibition HDAC4: 89.9 |
|  |  |  |
| MC2129  % inhibition HDAC1: 78.1  % inhibition HDAC4: 92.8 | MC1857  % inhibition HDAC1: 79.3  % inhibition HDAC4: 42.2 | MC1864  % inhibition HDAC1: 55.0  % inhibition HDAC4: 60.4 |
|  |  |  |
| MC2074  % inhibition HDAC1: 72.6  % inhibition HDAC4: 35.8 | MC1895  % inhibition HDAC1: 72.5  % inhibition HDAC4: 16.1 | MC1738  IC_50_ HD1-B: 4.6 nM  IC_50_ HD1-A: 4.4 nM |
|  |  |  |
| MC1924  IC_50_ HD1-B: 55 nM  IC_50_ HD1-A: 59 nM | MC1925  IC_50_ HD1-B: 24 nM  IC_50_ HD1-A: 29 nM | MC1930  IC_50_ HD1-B: 60 nM  IC_50_ HD1-A: 338 nM |
|  |  |  |
| MC1931  IC_50_ HD1-B: 62 nM  IC_50_ HD1-A: 325 nM | MC2709  Ref. 7 | MC2711  Ref. 7 |
|  |  |  |
| SAHA  % inhibition HDAC1: 80.8  % inhibition HDAC4: 76.3 | MS-275  % inhibition HDAC1: 65.4  % inhibition HDAC4: 0 |  |

Synthetic procedures used to obtain the unpublished compounds as well as their chemical and physical data will be published elsewhere.

Cell-based human HDAC1 and HDAC4 assays have been performed on human U937 (HDAC1) or ZR75.1 (HDAC4) cells using the specific antibodies (Abcam for HDAC1 and Sigma for HDAC4) according to the published procedure [5]. *In vitro* maize HD1-B and HD1-A enzyme inhibition assays have been carried out as previously reported [2-4].

**References.**

[1] [Mai A, Massa S, Ragno R, Cerbara I, Jesacher F, Loidl P, Brosch G.](http://www.ncbi.nlm.nih.gov/pubmed/12570373?ordinalpos=57&itool=EntrezSystem2.PEntrez.Pubmed.Pubmed_ResultsPanel.Pubmed_DefaultReportPanel.Pubmed_RVDocSum) 3-(4-Aroyl-1-methyl-1H-2-pyrrolyl)-N-hydroxy-2-alkylamides as a new class of synthetic histone deacetylase inhibitors. 1. Design, synthesis, biological evaluation, and binding mode studies performed through three different docking procedures. *J Med Chem*. 2003, **46**:512-524.

[2] [Mai A, Valente S, Rotili D, Massa S, Botta G, Brosch G, Miceli M, Nebbioso A, Altucci L.](http://www.ncbi.nlm.nih.gov/pubmed/17482499?ordinalpos=18&itool=EntrezSystem2.PEntrez.Pubmed.Pubmed_ResultsPanel.Pubmed_DefaultReportPanel.Pubmed_RVDocSum) Novel pyrrole-containing histone deacetylase inhibitors endowed with cytodifferentiation activity.

*Int J Biochem Cell Biol*. 2007, **39**:1510-1522.

[3] [Mai A, Massa S, Pezzi R, Rotili D, Loidl P, Brosch G.](http://www.ncbi.nlm.nih.gov/pubmed/14584932?ordinalpos=54&itool=EntrezSystem2.PEntrez.Pubmed.Pubmed_ResultsPanel.Pubmed_DefaultReportPanel.Pubmed_RVDocSum) Discovery of (aryloxopropenyl)pyrrolyl hydroxyamides as selective inhibitors of class IIa histone deacetylase homologue HD1-A**.** *J Med Chem*. 2003, **46**:4826-4829.

[4] [Mai A, Massa S, Pezzi R, Simeoni S, Rotili D, Nebbioso A, Scognamiglio A, Altucci L, Loidl P, Brosch G.](http://www.ncbi.nlm.nih.gov/pubmed/15857140?ordinalpos=41&itool=EntrezSystem2.PEntrez.Pubmed.Pubmed_ResultsPanel.Pubmed_DefaultReportPanel.Pubmed_RVDocSum) Class II (IIa)-selective histone deacetylase inhibitors. 1. Synthesis and biological evaluation of novel (aryloxopropenyl)pyrrolyl hydroxyamides**.** *J Med Chem*. 2005, **48**:3344-3353.

[5] [Mai A, Massa S, Rotili D, Simeoni S, Ragno R, Botta G, Nebbioso A, Miceli M, Altucci L, Brosch G.](http://www.ncbi.nlm.nih.gov/pubmed/17004718?ordinalpos=27&itool=EntrezSystem2.PEntrez.Pubmed.Pubmed_ResultsPanel.Pubmed_DefaultReportPanel.Pubmed_RVDocSum) Synthesis and biological properties of novel, uracil-containing histone deacetylase inhibitors*. J Med Chem*. 2006, **49**:6046-6056.

[6] [Mai A, Perrone A, Nebbioso A, Rotili D, Valente S, Tardugno M, Massa S, De Bellis F, Altucci L.](http://www.ncbi.nlm.nih.gov/pubmed/18381238?ordinalpos=8&itool=EntrezSystem2.PEntrez.Pubmed.Pubmed_ResultsPanel.Pubmed_DefaultReportPanel.Pubmed_RVDocSum) Novel uracil-based 2-aminoanilide and 2-aminoanilide-like derivatives: histone deacetylase inhibition and in-cell activities. *Bioorg Med Chem Lett*. 2008, **18**:2530-2535.

[7] [Moradei OM, Mallais TC, Frechette S, Paquin I, Tessier PE, Leit SM, Fournel M, Bonfils C, Trachy-Bourget MC, Liu J, Yan TP, Lu AH, Rahil J, Wang J, Lefebvre S, Li Z, Vaisburg AF, Besterman JM.](http://www.ncbi.nlm.nih.gov/pubmed/17941625?ordinalpos=7&itool=EntrezSystem2.PEntrez.Pubmed.Pubmed_ResultsPanel.Pubmed_DefaultReportPanel.Pubmed_RVDocSum) Novel aminophenyl benzamide-type histone deacetylase inhibitors with enhanced potency and selectivity. *J Med Chem*. 2007, **50**:5543-5546.
