# Supplementary material for: "Shock and kill" effects of class I-selective histone deacetylase inhibitors in combination with the glutathione synthesis inhibitor buthionine sulfoximine in cell line models for HIV-1 quiescence
Source: Retrovirology. 2009 Jun 2;6:52. doi: 10.1186/1742-4690-6-52 (PMC2697151; doi:10.1186/1742-4690-6-52)
Supplement: Additional file 2 — To study the HDACI response in a cell population, we used quiescently infected T-lymphoid Jurkat cell clones. Two types of cell clones were used: 1) A1, and A2, which have an integrated GFP/Tat construct under control of the HIV-1 LTR; 2) 6.3, and 8.4, which contain the entire HIV-1 genome under control of the LTR and have the GFP gene replacing nef. The 6.3 cells display insignificant basal levels of GFP expression. Cells were incubated with the different treatments, and GFP expression was monitored in gated live cells at 12, 24 and 72 hours by standard flow cytometric techniques. Results are presented as fluorescence histograms. Each histogram reports the percentage of fluorescent cells beyond a threshold value established using non-infected Jurkat cells. [file 1742-4690-6-52-S2.ppt]

## Slide 1
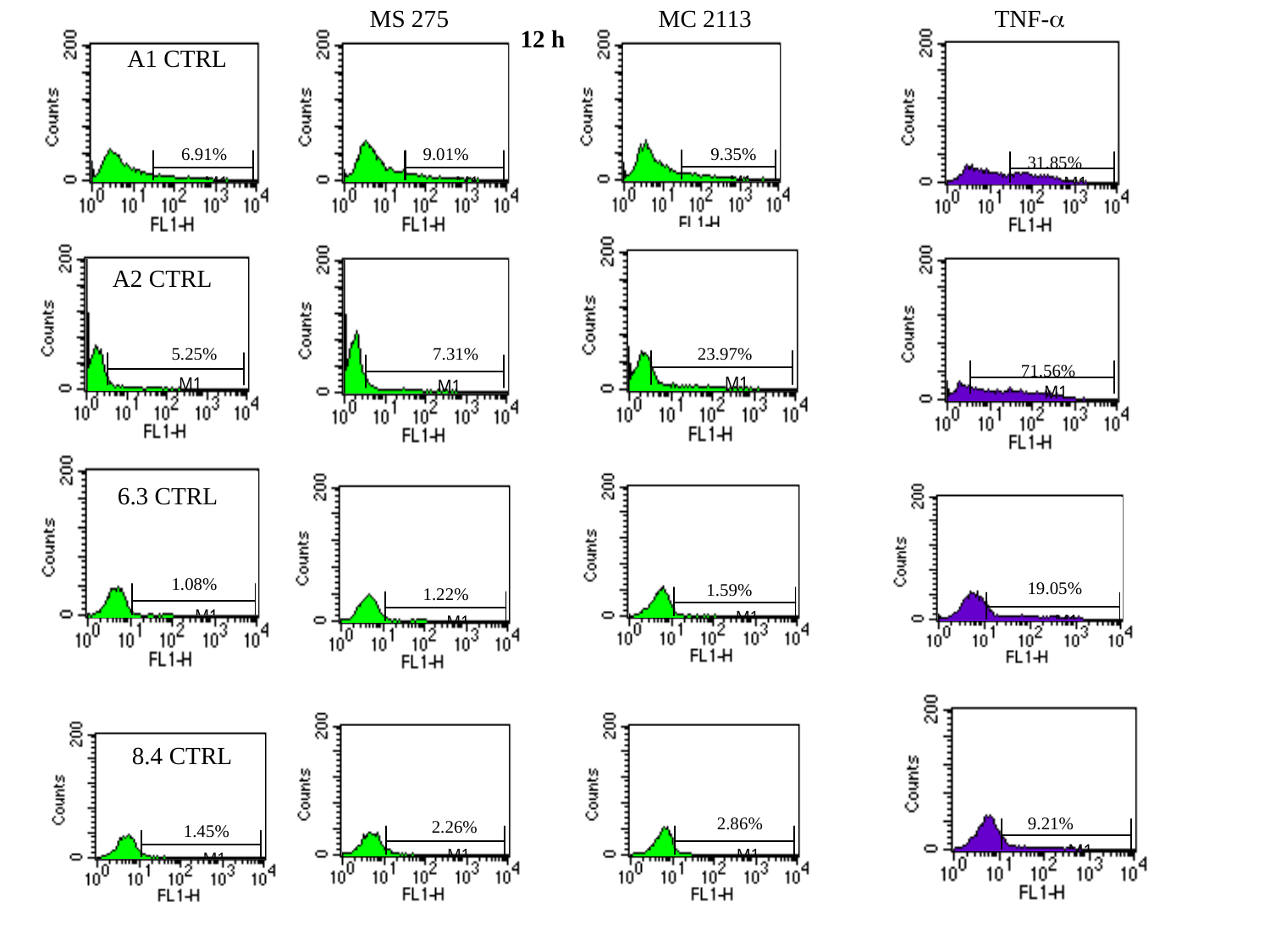

MS 275
MC 2113
TNF-α
 12 h
A1 CTRL
6.91%
9.01%
9.35%
31.85%
A2 CTRL
5.25%
7.31%
23.97%
71.56%
6.3 CTRL
1.08%
19.05%
1.59%
1.22%
8.4 CTRL
2.86%
 9.21%
2.26%
1.45%

## Slide 2
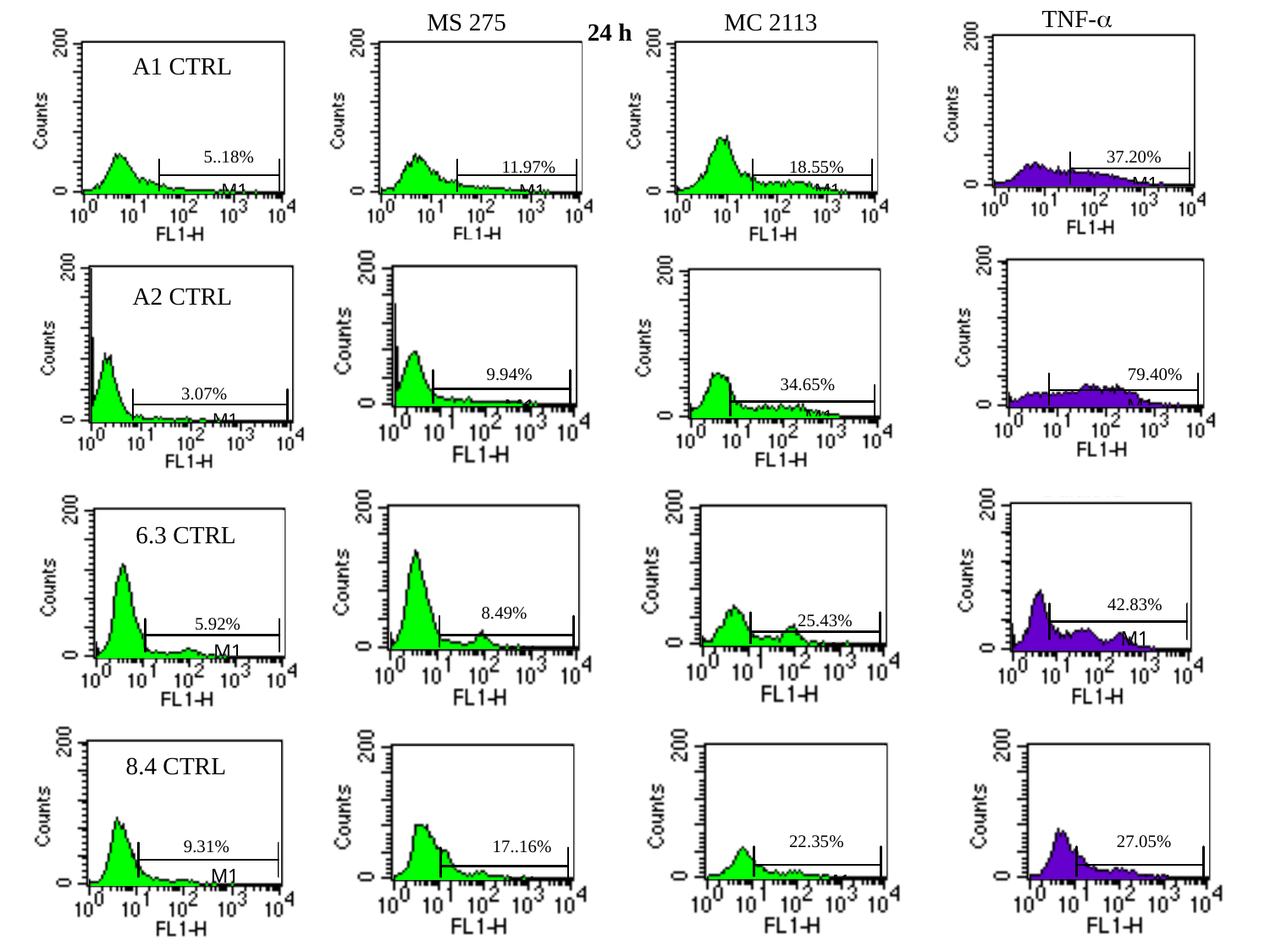

MS 275
MC 2113
TNF-α
24 h
A1 CTRL
5..18%
37.20%
11.97%
18.55%
A2 CTRL
9.94%
79.40%
34.65%
3.07%
6.3 CTRL
42.83%
8.49%
25.43%
5.92%
8.4 CTRL
22.35%
27.05%
9.31%
17..16%

## Slide 3
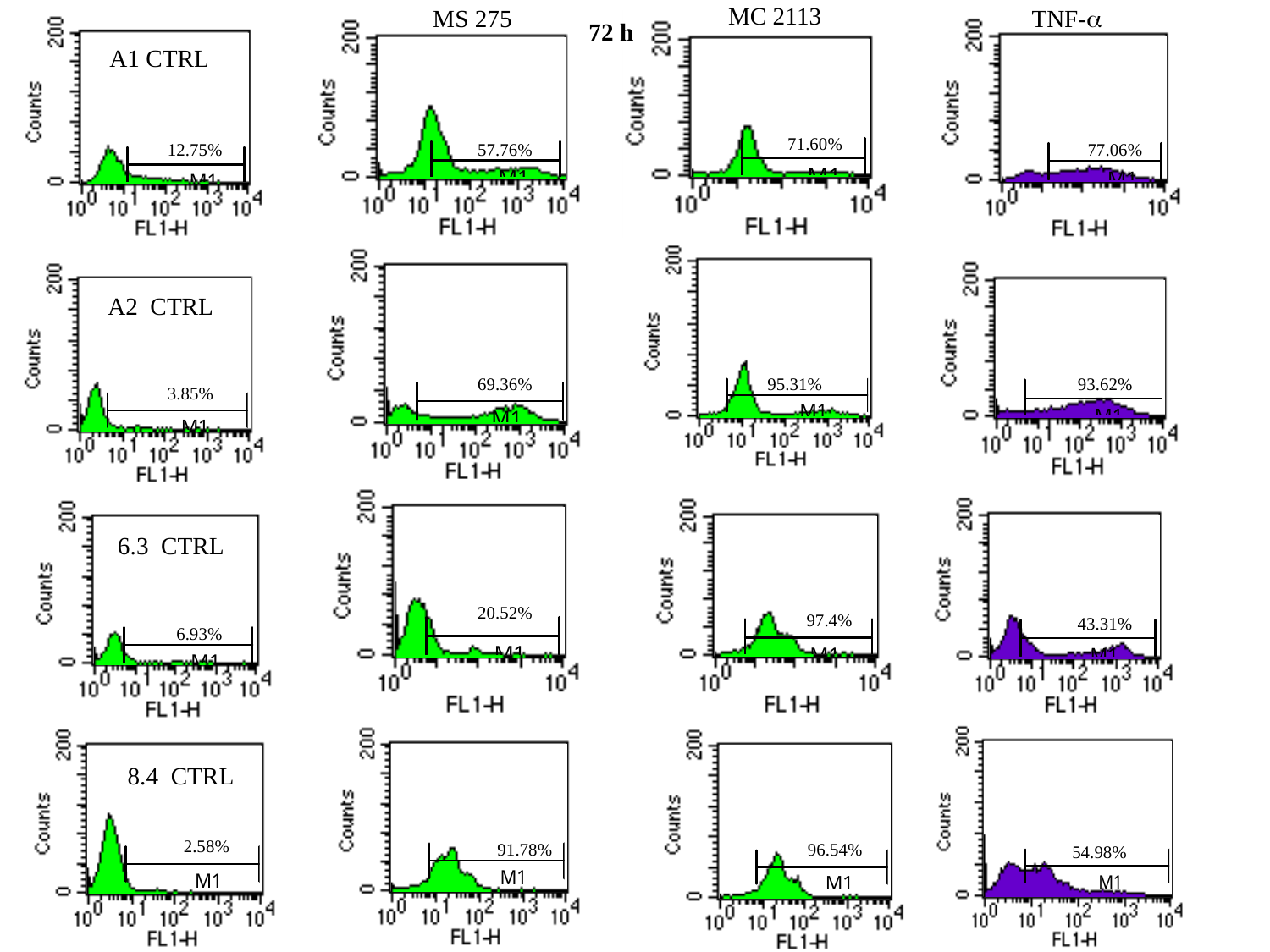

MC 2113
MS 275
TNF-α
72 h
A1 CTRL
71.60%
12.75%
57.76%
77.06%
A2 CTRL
69.36%
95.31%
93.62%
3.85%
6.3 CTRL
20.52%
97.4%
43.31%
6.93%
8.4 CTRL
2.58%
91.78%
96.54%
54.98%
