# Supplementary figures and images for: "Shock and kill" effects of class I-selective histone deacetylase inhibitors in combination with the glutathione synthesis inhibitor buthionine sulfoximine in cell line models for HIV-1 quiescence
Source: Retrovirology. 2009 Jun 2;6:52. doi: 10.1186/1742-4690-6-52 (PMC2697151; doi:10.1186/1742-4690-6-52)

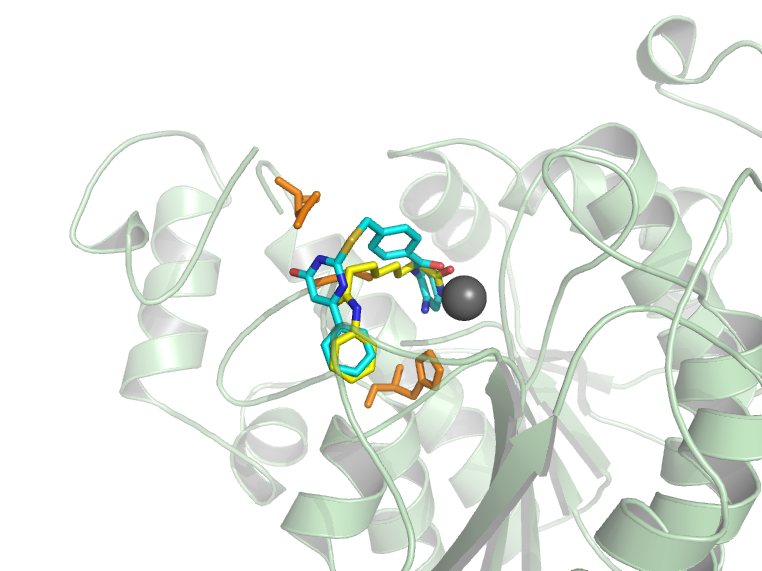

Supplement: Additional file 3 — Structural superimposition of MC2211 (carbon backbone in cyan) and SAHA (vorinostat; carbon backbone in yellow) docking at the HDAC2 catalytic site. SAHA, a non-selective HDACI, displays an amide group in a conformation that does not match that of the class I-selective HDACIs (Figure 3). The other molecular players are displayed in the same fashion as in Figure 3. [file 1742-4690-6-52-S3.png]
